# Phase-Specific Variations in Lower-Limb Muscle Strength Across the Menstrual Cycle in Female Soccer Players

**DOI:** 10.3390/sports14070257

**Published:** 2026-06-23

**Authors:** Christina Sefotha, Simoné Ferreira, Lynn Smith

**Affiliations:** Department of Sport and Movement Studies, Faculty of Health Sciences, University of Johannesburg, Johannesburg 2028, South Africa; simonef@uj.ac.za (S.F.); lynnvr@uj.ac.za (L.S.)

**Keywords:** menstrual cycle, menstruation phase, strength, late follicular phase, luteal phase, females, soccer

## Abstract

Fluctuations in ovarian hormones across the menstrual cycle (MC) have been suggested to influence neuromuscular performance in female athletes. However, phase-specific variations in lower-limb muscle strength remain underexplored, particularly within the soccer population. This study investigated phase-related differences in lower-limb muscle strength across MC phases in female soccer players. A repeated-measures design was employed involving 50 competitive female soccer players. Bilateral lower-limb muscle strength was assessed using a handheld dynamometer (VALD DynaMo Plus) during the three MC phases: menstruation, late follicular, and luteal phase. Estimated menstrual cycle phases were identified using calendar-estimated tracking or an MC monitoring application (FitrWoman). Phase-related differences were analysed using repeated-measures analysis of variance with Bonferroni-adjusted post hoc comparisons, and effect sizes were reported as partial eta squared (ηp^2^). Significant differences in lower-limb muscle strength were observed across estimated MC phases (*p* < 0.05, ηp^2^ = 0.12–0.31). Both the non-dominant and dominant limbs demonstrated higher strength values during the late follicular phase, with hip abductors emerging as the strongest muscle group bilaterally (≈149 ± 37 kg). Most muscle groups exhibited lower strength values during the menstruation phase. Lower-limb muscle strength appears to vary across calendar-estimated MC phases in female soccer players, with higher strength values observed during the late follicular phase and lower values during menstruation. These findings should be interpreted with caution due to the method of phase identification but may have implications for the scheduling of strength assessments and training load management in female athletes.

## 1. Introduction

The increasing participation and elite performance of female athletes have led to growing recognition of sex-specific considerations in sports science [1]. While enhanced female involvement in competitive sport has contributed to improved performance standards, it has also been accompanied by increased incidence of sports-related injuries [2]. As a result, research attention has progressively shifted toward female-specific physiology and its implications for exercise performance and injury risk [3]. Despite this advancement, evidence examining the influence of the menstrual cycle (MC), a complex and dynamic physiological process, on intrinsic injury risk factors remains limited. Historically, MC-related research focused primarily on menstrual dysfunction within the female athlete triad [4]; however, more recent literature has begun to explore the independent effects of MC phases on athletic performance outcomes [5]. Further investigation is therefore required to clarify how MC phases influence modifiable injury risk factors, particularly muscle strength, which plays a critical role in both performance and injury risk assessment [6].

Female soccer players are disproportionately affected by lower-extremity injuries, which account for approximately 85% of all reported injuries, with nearly half resulting from non-contact mechanisms [7]. Injury-related time loss has been reported to be approximately 21% higher in female compared with male soccer players, largely due to an increased incidence of severe knee and ankle ligament injuries, including anterior cruciate ligament (ACL) injuries that occur two to eight times more frequently in females [8]. This elevated injury risk has been attributed to sex-specific factors such as neuromuscular control patterns and hormonal fluctuations associated with the MC [9]. Injury risk factors in sport are commonly categorised as extrinsic or intrinsic, with intrinsic factors further classified as modifiable or non-modifiable. While non-modifiable intrinsic risk factors include age, sex, and injury history, muscle strength represents a key modifiable intrinsic factor and remains central to injury prevention research in female athletes [10].

Muscular strength is defined as the ability to exert force against an external resistance or to overcome an opposing force [11]. Higher levels of muscular strength are associated with improved athletic performance and reduced injury risk [12]. A systematic review [12] demonstrated that increased muscular strength is associated with enhanced performance in fundamental sport-related tasks, including jumping, sprinting, and change in direction ability. Evidence further suggests that greater lower-limb strength contributes to improved sprint performance and reduced susceptibility to lower extremity injuries [12]. Insufficient strength in the quadriceps, hamstrings, or hip musculature has been associated with an increased risk of knee and ACL injuries [13]. However, findings across existing studies remain inconsistent, and much of the available evidence is derived from adult or mixed-sex populations, limiting its applicability to female soccer players specifically [13]. However, the interpretation of MC-related effects on performance is complicated by substantial inter- and intra-individual variability in cycle characteristics, as well as challenges associated with accurate phase identification in applied research settings.

The concentration of female sex hormones has been proposed to be associated with variations in neuromuscular performance, potentially altering force production and muscle activation patterns [14]. It has been hypothesised that lower progesterone concentrations during the follicular phase, particularly when estrogen levels peak in the late follicular phase, may be associated with enhanced strength and power outcomes [14]. Previous studies have reported phase-dependent variations in lower-limb muscle strength, with reduced strength observed during the early follicular phase [15] and altered neuromuscular characteristics during the ovulatory and luteal phase [16]. The luteal phase has been associated with decreased ankle strength, increased joint laxity, and altered lower extremity muscle activity, potentially increasing injury risk in female athletes [17].

Despite emerging evidence suggesting MC-related variations in neuromuscular performance, findings remain inconsistent, and limited research has examined phase-specific changes in lower-limb muscle strength in female soccer players, particularly within applied, field-based settings. Therefore, the aim of this study was to investigate phase-related differences in lower-limb muscle strength across MC phases in female soccer players competing at various soccer clubs in Johannesburg, South Africa.

## 2. Materials and Methods

### 2.1. Research Design and Study Site

A repeated-measures research design was employed to examine phase-specific variations in lower-limb muscle strength across the MC in female soccer players. Each participant underwent strength assessments during three distinct estimated MC phases: menstruation, late follicular, and luteal phase. The identified MC phases represent estimated temporal windows and not hormonally verified MC phases. This design allowed for within-subject comparisons across phases, thereby reducing inter-individual variability.

The study was conducted at selected soccer clubs within the Johannesburg metropolitan area, South Africa. All testing procedures were performed in a controlled environment at the respective club training facilities to ensure consistency across testing sessions. Data collection took place during the competitive season, with testing sessions scheduled to minimise disruption to regular training routines.

### 2.2. Participants, Selection and Recruitment

The study sample consisted of 50 female soccer players aged 18 years and older, competing at various competitive levels within soccer clubs in Johannesburg, South Africa. Soccer players were selected as the target population due to the lower-limb dominant nature of the sport. Participants were recruited from the University of Johannesburg Women’s Football Club and Kempton Park Ladies Football Club.

A convenience sampling approach was used. Permission to recruit players was obtained from club owners, managers, or head coaches prior to participant recruitment. Following institutional approval, eligible players were invited to participate and were provided with detailed information regarding the study procedure. Written informed consent was obtained from all participants prior to data collection.

Participants’ recruitment commenced in February 2024. Strength testing was conducted between May 2024 and June 2025, with the first assessment completed on 16 May 2024 and the final assessment on 7 June 2025.

#### Inclusion and Exclusion Criteria

Participants were eligible for inclusion if they were female soccer players aged 18 years or older, actively competing at club level, and had a self-reported regular MC. Participants were required to be free from musculoskeletal injury at the time of testing and able to complete all strength assessments across the three designated estimated MC phases.

Participants were excluded if they reported a current injury or medical condition that could affect lower-limb muscle strength, had undergone recent lower-limb surgery, or were unable to complete testing during all estimated MC phases. Additional exclusion criteria included pregnancy or the use of medication known to influence neuromuscular performance.

### 2.3. Testing Procedure

#### 2.3.1. Tracking the MC

Estimated MC phases were tracked using the FitrWoman application (version 2.9.20; Orecco Ltd., Twickenham, UK), which is a validated, calendar-estimated MC monitoring tool [18,19]. Participants were required to download the application onto their personal Android or iOS smart devices and log the onset and duration of menstruation. The application automatically classified estimated MC phases based on individual cycle length, allowing identification of the menstruation, late follicular, and luteal phases. The MC phases are divided as follows on the Fitrwoman application: Phase 1 was from the first to the last day of bleeding (menstruation), phase 2 is from when the bleeding stops to the mid-point of the cycle (late follicular phase), phase 3 is from the mid-point of the cycle to the last 5 days and phase 4 (pre-menstruation) is from the last 5 days of the cycle (phase 3 and 4 are the luteal phase).

Participants were instructed to record their MC data from the onset of menstruation through to the end of the luteal phase and continue tracking until all strength assessments had been completed across the designated phases. For participants who did not have access to a compatible smart device, a manual calendar-estimated tracking method was used. The menstruation phase was recorded from day 1 to 6, the late follicular phase was from day 7 to 14 and the luteal phase was from day 15 to 28. These participants reported the start and end dates of menstruation for two consecutive cycles, enabling phase identification based on cycle length and timing.

Menstrual cycle phase identification was based on calendar-derived estimations rather than biochemical verification (e.g., hormonal assays or ovulation testing). The identified MC phases in the study are a representation of estimated temporal windows. While this approach is commonly used in applied field-based research, it may introduce potential misclassification due to inter- and intra-individual variability in cycle length and ovulation timing. As a result, phase-specific interpretations should be considered with caution.

#### 2.3.2. Muscle Strength Measurement

Lower-limb muscle strength was assessed bilaterally using a handheld dynamometer (VALD DynaMo Plus, VALD Performance, Brisbane, Australia). This device has been shown to provide valid and reliable measurements of isometric muscle strength in athletic populations. The VALD DynaMo Plus demonstrated excellent reliability for isometric strength measurements, with reported within-session and between-session intraclass correlation coefficients (ICC) ranging from 0.90 to 0.99 across upper- and lower-limb isometric tests, indicating very high measurement consistency [20]. In terms of validity, isometric force values obtained using the DynaMo Plus showed strong concurrent validity when compared with criterion dynamometry, with correlation coefficients of r ≥ 0.90 and low measurement error, supporting its accuracy for assessing isometric strength in healthy adults [20]. All assessments were conducted by the same trained researcher to minimise inter-rater variability; however, no study-specific intra-rater reliability analysis was conducted.

Strength testing was performed for key lower-limb muscle groups relevant to soccer performance and injury risk, including hip flexors, hip extensors, hip abductors, hip adductors, hip internal and external rotators, knee flexors, knee extensors, ankle dorsiflexors and plantar flexors. Multiple muscle groups were included to reflect the integrated contribution of the lower-limb kinetic chain to soccer performance and injury risk. Assessing both dominant and non-dominant limbs allowed for the evaluation of potential asymmetries and limb-specific adaptations relevant to sport-specific tasks. Standardised testing positions and stabilisation techniques were used in accordance with manufacturer guidelines and previous published protocols. Before testing, a researcher-led warm-up was conducted using a cycle ergometer for five minutes (60 Watts), to minimise any risks of injury during testing. Participants performed maximal voluntary isometric contractions for each muscle group, with verbal encouragement provided to ensure maximal effort.

For each muscle group, participants completed three maximal trials per limb, with a standardised rest period between trials to minimise fatigue. The highest recorded value for each muscle group and limb was used for analysis to reflect peak maximal voluntary contraction, consistent with standard strength assessment protocols. Strength values were recorded in kilograms (kg). Strength values represent kilogram-force equivalents as provided by the device software. All testing sessions were conducted under consistent conditions across estimated MC phases to ensure comparability of measurements.

A fixed testing order was used across all participants to ensure consistency between sessions. However, the absence of randomisation or counterbalancing may have introduced potential fatigue or order effects.

Testing sessions were scheduled to minimise external influences, with assessments conducted either on rest days or prior to training sessions where possible. However, factors such as training load, fatigue, nutritional status, and time of day were not formally controlled and may have influenced strength outcomes.

### 2.4. Statistical Analysis

Statistical analyses were performed using IBM SPSS Statistics (version 28; IBM Corp., Armonk, NY, USA). Descriptive statistics, including means and standard deviations, were calculated for all strength variables. Data were screened for normality using the Shapiro–Wilk test.

Phase-specific differences in lower-limb muscle strength were analysed using a one-way repeated-measures analysis of variance (ANOVA) with multivariate tests, with estimated MC phases (menstruation, late follicular, luteal) as the within-subject factor. The use of multivariate tests was appropriate due to the inclusion of multiple related dependent variables across joints and movements, allowing for the assessment of overall phase-related effects while reducing the likelihood of Type I error.

Where significant main effects were identified, Bonferroni-adjusted post hoc comparisons were conducted to determine pairwise differences between phases. Effect sizes were calculated using partial eta squared (ηp^2^) and interpreted as small (0.01), moderate (0.06), or large (≥0.14). Statistical significance was set at *p* < 0.05, with effect sizes considered to support interpretation of practical relevance.

Given the number of dependent variables analysed across multiple joint movements and limb conditions, there is an increased risk of Type I error. To address this, variables were conceptually grouped by joint (hip, knee, ankle) and findings were interpreted based on consistency of patterns across related muscle groups rather than isolated statistically significant results.

### 2.5. Ethical Considerations

The study was conducted in accordance with the Declaration of Helsinki. Ethical approval was obtained from the University of Johannesburg Research Ethics Committee (REC-2369-2023). Permission was obtained from the relevant soccer clubs prior to participant recruitment. All participants provided written informed consent before participation and were informed of their right to withdraw from the study at any time without any repercussions.

## 3. Results

Participant characteristics

A total of 50 female soccer players were included in the study. Participant demographics and MC characteristics are summarised in Table 1. The mean age of the participants was 21.68 ± 3.24 years, with the mean height of 1.60 ± 0.08 m and a mean body mass of 56.62 ± 10.32 kg.

The majority of participants reported right-leg dominance (n = 40; 80.0%), while 10 participants (20%) reported left-leg dominance. With regard to MC type, 37 participants (74.0%) reported eumenorrheic cycles, 13 participants (26.0%) reported oligomenorrheic cycles, and no participants were classified as polymenorrheic (a MC interval of less than 21 days). All participants completed strength assessments across the three designated estimated MC phases, and no data were excluded due to injury or incomplete testing. Given the large variation in body mass within the sample (41–82 kg), non-normalized strength measurements may alter the interpretation of between-participant variability.

Lower-limb muscle strength across MC phases

Mean lower-limb muscle strength values for the dominant (DL) and non-dominant (NDL) limbs across the menstruation, late follicular, and luteal phases are presented in Table 2. Across most muscle groups, strength values differed across MC phase, with higher values generally observed during the late follicular phase and lower values during the menstruation phase. Strength values during the luteal phase were typically intermediate.

No consistent differences were observed between the DL and NDL, with similar phase-related patterns evident bilaterally across muscle groups.

Across both limbs, the hip abductors demonstrated the highest strength values, with peak values observed during the late follicular phase (≈148–149 kg). Lower strength values were most commonly observed during the menstruation phase across most muscle groups. Some variation was noted between movements, with hip extensors and knee flexors demonstrating lower values during the luteal phase in specific instances. A 95% confidence interval was used with a *p*-value of <0.05.

Statistical comparison of lower-limb muscle strength across MC phases

Phase-specific differences in lower-limb muscle strength were analysed using repeated-measures analysis of variance (ANOVA) with multivariate tests, with estimated MC phases (menstruation, late follicular, luteal) as the within-subject factor. Wilks’ lambda values, associated F-statistics, *p*-values, and partial eta squared (ηp^2^) effect sizes for each joint movement are presented in Table 3. Effect sizes were interpreted as small (ηp^2^ = 0.01), moderate (ηp^2^ = 0.06), and large (ηp^2^ ≥ 0.14). A 95% confidence interval was used with a *p*-value of <0.05.

Significant differences in muscle strength across estimated MC phases were observed for most joint movements (*p* < 0.05). Detailed statistical outcomes for all assessed movements are presented in Table 3. These effects were identified across hip movements, knee extension, and ankle dorsiflexion and plantarflexion. No statistically significant differences were observed for knee flexion (*p* = 0.585). Effect size analysis indicated moderate to large effects for most movements (ηp^2^ ≥ 0.14), with the largest effect observed for the hip adduction (ηp^2^ = 0.284). Supplementary tables (Tables S1 and S2) with the full post hoc tests from the pairwise comparisons for the non-dominant leg and dominant leg throughout the three phases were provided. A 95% confidence interval was used with a *p*-value of <0.05.

**Figure 1:**
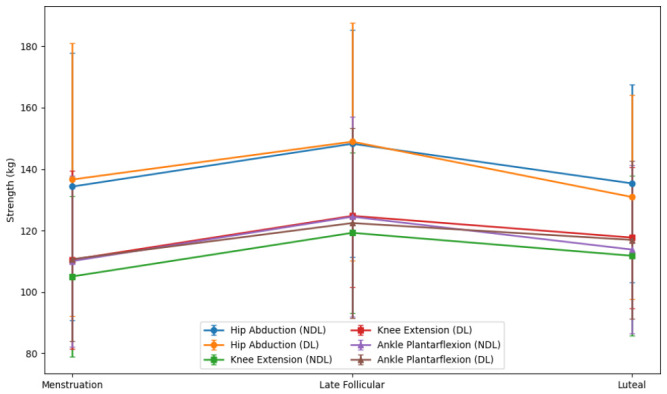
Mean (± SD) lower-limb muscle strength for the dominant (DL) and non-dominant (NDL) limbs across estimated MC phases for hip abduction, knee extension, and ankle plantarflexion.

Phase-related changes in key lower-limb strength measures across the MC are illustrated in Figure 1.

Figure 1 presents mean (±SD) lower-limb muscle strength across MC phases for selected movements. Similar phase-related patterns were observed across both the dominant and non-dominant limbs. The findings presented in the study do not represent evidence of direct hormonal impacts because the indicated phases are estimated temporal windows rather than hormonally verified MC phases.

## 4. Discussion

The purpose of this study was to examine phase-specific variations in lower-limb muscle strength across the MC in female soccer players. The main findings indicate that muscle strength differed across estimated MC phases for most assessed movements, with higher strength values generally observed during the late follicular phase and lower values during menstruation. These findings were evident across both DL and NDL and were supported by moderate to large effect sizes for the majority of movements.

Menstrual cycle phases and muscle strength

The present study demonstrated that peak lower-limb strength generally occurred during the late follicular phase, particularly in the knee flexors and extensors. This phase coincides with rising estrogen concentrations, which have been associated with variations in neuromuscular performance in the previous literature. These findings are broadly consistent with previous research reporting variations in quadriceps and hamstring strength across estimated MC phases, when estrogen levels are elevated [21]. Conversely, reduced strength values were more evident during the early follicular and luteal phases. The early follicular phase is characterised by low concentrations of both estrogen and progesterone, while the luteal phase involves elevated progesterone levels, which have been suggested to be associated with changes in neuromuscular performance. Although not all literature reports consistent phase-related differences in knee strength, the current findings which are based on estimated temporal windows of the MC phases support the growing perspective that hormonal fluctuations may influence maximal force production in a phase-dependent manner.

Movement-specific strength responses

A key observation from this study is that MC–related strength variations were movement-specific. The hip abductors demonstrated the greatest strength during the late follicular phase bilaterally, suggesting that lateral stabilisers may exhibit phase-related variation. Given the demands of soccer, including lateral displacement, pelvic stability and single-leg control, well-developed hip abductors are essential for both performance and injury prevention. Interestingly, hamstring strength did not follow a uniform phase-related pattern. While some literature reports reduced hamstring-to-quadriceps ratios during the early follicular phase, the present study identified the hamstrings as the weakest during the luteal phase on the DL. These findings suggest that strength responses across the MC may differ depending on contraction type, joint action and limb dominance. These movement-specific findings reinforce the notion that MC influences should not be generalised across all muscle groups. Instead, hormonal effects based on the estimated temporal windows of the MC phases may interact with neuromuscular recruitment strategies, joint mechanics and sport-specific loading patterns, resulting in differential strength expression.

Dominant and non-dominant limb considerations

Limb dominance remains a significant characteristic in soccer performance, given the sport’s reliance on unilateral lower-limb actions such as kicking, passing and directional changes [22]. In the present study, hip and knee muscle groups demonstrated greater strength on the DL, whereas ankle strength was higher on the NDL. These findings may reflect task-specific adaptations associated with soccer participation, where the DL is frequently used for force production, such as kicking, while the NDL contributes substantially to stabilisation and support. Strength asymmetries and lateral dominance are still debatable topics in soccer despite the abundance of literature on the subject. Numerous studies have shown that the dominant limb is significantly stronger and more powerful than the NDL, while other research has asserted that there is no discernible difference between the two legs in soccer [23].

Practical implications for training and injury risk management

Understanding phase-related and limb-specific strength patterns has practical relevance for female soccer players. Given the importance of hip abductor strength for pelvic control, dynamic balance, and change-of-direction tasks, maintaining optimal strength in this muscle group is critical for both performance enhancement and injury prevention. The observation of greater strength during the late follicular phase suggests that this phase may be associated with higher strength values; however, this should be interpreted with caution. However, given the variability in strength expression across estimated MC phases and between limbs, rigid MC–based programming may not be appropriate. Instead, practitioners may consider adopting an individualised monitoring approach, incorporating unilateral strength assessments to identify asymmetries and phase-related fluctuations.

Targeted strengthening of weaker muscle groups—particularly hamstrings during phases of reduced torque—may contribute to improved hamstring-to-quadriceps balance; however, this relationship was not directly assessed in the present study. Additionally, monitoring dominant and non-dominant limb differences can assist in designing corrective interventions aimed at minimising maladaptive asymmetries. Overall, the findings emphasise that lower-limb strength in female soccer players is influenced by both limb dominance and estimated MC phase in a muscle-specific manner. Integrating these considerations into training design may enhance performance optimisation while supporting injury risk management strategies. The inclusion of multiple dependent variables across joint movements and limb dominance increases the analytical complexity of the study and the potential for Type I error. Although effect sizes were presented to assist in interpreting the findings, the independence of statistical tests may be limited due to the biomechanical interdependence of lower-limb joints. To overcome this, findings were interpreted in terms of phase-related patterns among muscle groups rather than separate statistically significant outcomes. Nevertheless, the possibility of false-positive findings cannot be excluded and should be considered when interpreting the results.

However, given the use of calendar-estimated MC tracking and the observational design of the study, these findings should not be used to prescribe MC-based training interventions without further research.

Limitations and future research

Estimated MC phases were identified using calendar-estimated tracking rather than biochemical hormone confirmation, which may introduce variability in phase identification. Reliance on calendar-estimated MC phases and oligomenorrheic participants may lead to misclassification, thereby attenuating actual phase-specific differences in muscular strength, given the significant inter- and intra-individual heterogeneity in cycle duration. A minority of the participants who reported contraceptive use were excluded from the study which limited comparisons from participants who use hormonal contraceptives and non-users. This problem significantly reduces the confidence in attributing variations in strength to certain hormonal settings.

Additionally, the observational design precludes causal inference, and injury outcomes were not assessed. The study’s sample characteristics, which included female soccer players from selected Johannesburg clubs, may also restrict the generalisability of the findings. The extent to which these findings may be applied to athletes in different sports, geographical areas, or performance contexts may be limited by variations in training load, competitive level, access to medical care, use of contraceptives, and cultural attitudes toward menstrual health.

Future research should incorporate hormonal verification, longitudinal monitoring across multiple cycles, and injury surveillance to further clarify the relationship between estimated MC phases, strength variability, and injury risk in female athletes.

## 5. Conclusions

The findings of this study demonstrate that lower-limb muscle strength in female soccer players differed across estimated MC phases, with strength values generally higher during the late follicular phase and lower during menstruation. These phase-related differences contribute to the growing body of literature suggesting that neuromuscular performance may vary across the MC in female athletes.

By identifying variability in strength across key lower-limb muscle groups, this study highlights the potential relevance of estimated MC phase as a contextual factor in performance monitoring. However, given the observational design and the use of calendar-estimated MC tracking, these findings should be interpreted with caution. Further research incorporating hormonal verification and longitudinal designs is required before practical application can be considered.

## Figures and Tables

**Table 1:** Participant demographic and menstrual cycle characteristics (n = 50).

Variable	Category/Statistic	Mean ± SD	Min	Max	n (%)
Age (years)	-	21.68 ± 3.24	18	32	-
Height (m)	-	1.60 ± 0.08	1.41	1.79	-
Body mass (kg)	-	56.62 ± 10.32	41.20	82.10	-
Dominant leg	Right	-	-	-	40 (80.0)
	Left	-	-	-	10 (20.0)
Menstrual cycle type	Eumenorrheic	-	-	-	37 (74.0)
	Oligomenorrheic	-	-	-	13 (26.0)

**Table 2:** Lower-limb muscle strength across menstrual cycle phases.

Joint	Movement	Limb	Menstruation	Late Follicular	Luteal
Hip	Flexion	NDL	120.28 ± 32.84	131.62 ± 31.05	123.00 ± 32.61
		DL	126.02 ± 28.40	137.10 ± 27.55	127.84 ± 25.99
	Extension	NDL	86.86 ± 26.57	96.40 ± 23.59	86.80 ± 25.75
		DL	85.04 ± 23.63	102.46 ± 24.17	89.92 ± 24.17
	Abduction	NDL	134.32 ± 43.42	148.26 ± 37.00	135.34 ± 32.20
		DL	136.62 ± 44.36	148.94 ± 38.65	130.90 ± 33.27
	Adduction	NDL	91.34 ± 33.69	106.36 ± 30.36	93.84 ± 29.32
		DL	94.68 ± 30.08	108.64 ± 25.95	96.24 ± 25.58
	Internal rotation	NDL	72.50 ± 21.23	80.32 ± 20.00	79.52 ± 16.94
		DL	71.82 ± 15.82	81.66 ± 19.58	79.72 ± 15.99
	External rotation	NDL	72.10 ± 19.69	82.32 ± 21.53	77.76 ± 23.09
		DL	74.54 ± 17.43	85.22 ± 22.21	81.02 ± 20.37
Knee	Flexion	NDL	94.08 ± 34.56	98.08 ± 25.38	94.64 ± 31.79
		DL	96.00 ± 34.49	108.56 ± 29.36	93.66 ± 24.68
	Extension	NDL	105.06 ± 26.23	119.26 ± 26.16	111.78 ± 25.96
		DL	110.54 ± 28.99	124.78 ± 23.17	117.70 ± 22.95
Ankle	Dorsiflexion	NDL	80.58 ± 24.97	92.66 ± 22.59	86.04 ± 22.56
		DL	80.62 ± 20.51	89.86 ± 19.35	86.50 ± 25.82
	Plantarflexion	NDL	110.06 ± 27.84	124.50 ± 32.45	113.80 ± 27.37
		DL	110.68 ± 26.72	122.44 ± 31.05	116.94 ± 25.70

Values are presented as mean ± standard deviation. Strength values are reported as kilogram-force equivalents.

**Table 3:** Statistical comparison of lower-limb muscle strength across menstrual cycle phases.

Joint	Movement	Wilks’ λ	F	df (hyp, err)	*p*-Value	ηp^2^
Hip	Flexion	0.850	4.23	2, 48	0.020 *	0.150 *
	Extension	0.818	5.35	2, 48	0.008 *	0.182 *
	Abduction	0.850	4.25	2, 48	0.020 *	0.150 *
	Adduction	0.716	9.50	2, 48	<0.001 *	0.284 *
	Internal rotation	0.831	4.90	2, 48	0.012 *	0.169 *
	External rotation	0.769	7.20	2, 48	0.002 *	0.231 *
Knee	Flexion	0.978	0.54	2, 48	0.585	0.022
	Extension	0.751	7.94	2, 48	0.001 *	0.249 *
Ankle	Dorsiflexion	0.800	6.00	2, 48	0.005 *	0.200 *
	Plantarflexion	0.752	7.91	2, 48	0.001 *	0.248 *

* Wilks’ lambda values are reported for multivariate tests. Effect sizes are presented as partial eta squared (ηp^2^).

## Data Availability

The data presented in this study are available on request from the corresponding author due to ensuring the anonymity of the participants is not compromised.

## Supplementary Materials

The following supporting information can be downloaded at: https://www.mdpi.com/article/10.3390/sports14070257/s1, Table S1: Muscle strength pairwise comparisons of the menstrual cycle (MC) phases on the non-dominant leg (NDL); Table S2: Musculoskeletal strength pairwise comparisons of the menstrual cycle (MC) phases on the dominant leg (DL).

## Abbreviations

The following abbreviations are used in this manuscript:

MC	Menstrual Cycle
kg	kilograms
DL	Dominant Leg
NDL	Non-Dominant Leg
