# Supplementary material for: Phase-Specific Variations in Lower-Limb Muscle Strength Across the Menstrual Cycle in Female Soccer Players
Source: Sports (Basel). 2026 Jun 23;14(7):257. doi: 10.3390/sports14070257 (PMC13418160; doi:10.3390/sports14070257)
Supplement: Supplementary file 1 [file sports-14-00257-s001.zip › sports-4273300-supplementary.pdf]

Supplementary Tables

Pairwise comparison tests: Muscle strength tests for the non-dominant leg and dominant leg throughout the three phases (full post hoc results)

Table S1: Muscle strength pairwise comparisons of the menstrual cycle (MC) phases on the non-dominant leg (NDL)

**Movement**	**Cycle phase**		**Mean difference**	**Std. error**	**Sig.**[b]	**95% confidence interval for difference**[b]	
						**Lower bound**	**Upper bound**
Hip flexion	2	1	11,340*	4,138	**0,026**	1,083	21,597
		3	8,620	4,619	0,204	−2,831	20,071
Hip extension	2	1	9,540*	3,517	**0,028**	0,821	18,259
		3	9,600*	3,466	**0,024**	1,008	18,192
Hip abduction	2	1	13,940	5,965	0,071	−0,848	28,728
		3	12,920*	4,677	**0,024**	1,326	24,514
	3	1	1,020	5,260	1,000	−12,020	14,060
		2	−12,920*	4,677	**0,024**	−24,514	−1,326
Hip adduction	2	1	15,020*	3,729	**0,001**	5,775	24,265
		3	12,520*	3,523	**0,003**	3,787	21,253
Hip internal rotation	2	1	7,820*	2,718	**0,018**	1,082	14,558
		3	0,800	2,401	1,000	−5,152	6,752
	3	1	7,020*	2,553	**0,025**	0,692	13,348
		2	−0,800	2,401	1,000	−6,752	5,152
Hip external rotation	2	1	10,220*	2,697	**0,001**	3,535	16,905
		3	4,560	2,894	0,365	−2,615	11,735
Knee extension	2	1	14,200*	3,527	**0,001**	5,456	22,944
		3	7,480	3,859	0,175	−2,086	17,046
Ankle plantarflexion	2	1	12,080*	3,451	**0,003**	3,525	20,635
		3	6,620	3,553	0,205	−2,187	15,427
Ankle dorsiflexion	2	1	14,440*	3,832	**0,001**	4,940	23,940

		3	10,700*	3,551	**0,012**	1,898	19,502

Based on estimated marginal means

* The mean difference is statistically significant at a level of ≤0.05

[b] Adjustment for multiple comparisons: Bonferroni

Sig = P-value

Table S2: Musculoskeletal strength pairwise comparisons of the menstrual cycle (MC) phases on the dominant leg (DL)

Movement	Cycle phase		Mean difference	Std. error	Sig.[b]	95% confidence interval for difference[b]	
						Lower bound	Upper bound
Hip flexion	2	1	11,080	4,503	0,052	−0,082	22,242
		3	9,260*	3,718	**0,049**	0,043	18,477
Hip extension	2	1	17,420*	2,953	**0,000**	10,099	24,741
		3	12,540*	3,568	**0,003**	3,695	21,385
Hip abduction	2	1	12,320	5,280	0,071	−0,769	25,409
		3	18,040*	4,444	**0,001**	7,025	29,055
Hip adduction	2	1	13,960*	4,226	**0,005**	3,483	24,437
		3	12,400*	3,664	**0,004**	3,316	21,484
Hip internal rotation	2	1	9,840*	2,524	**0,001**	3,583	16,097
		3	1,940	2,505	1,000	−4,270	8,150
	3	1	7,900*	2,498	**0,008**	1,708	14,092
		2	−1,940	2,505	1,000	−8,150	4,270
Hip external rotation	2	1	10,680*	2,780	**0,001**	3,787	17,573
		3	4,200	2,686	0,373	−2,458	10,858
Knee flexion	2	1	12,560*	4,784	**0,035**	0,701	24,419
		3	14,900*	3,589	**0,000**	6,002	23,798
Knee extension	2	1	14,200*	3,527	**0,001**	5,456	22,944
		3	7,480	3,859	0,175	−2,086	17,046
Ankle plantarflexion	2	1	9,240*	3,509	**0,034**	0,542	17,938
		3	3,360	3,721	1,000	−5,864	12,584

Based on estimated marginal means

* The mean difference is statistically significant at a level of ≤0.05

[b] Adjustment for multiple comparisons: Bonferroni

Sig= P-value
